# Outcomes of modular endoprosthesis reconstruction versus cement spacer reconstruction following resection of proximal humeral tumors

**DOI:** 10.1186/s12891-022-05432-4

**Published:** 2022-05-21

**Authors:** Walid Atef Ebeid, Sherif Eldaw, Ismail Tawfeek Badr, Mohamed Kamal Mesregah, Bahaa Zakarya Hasan

**Affiliations:** 1 Department of Orthopaedic Surgery, Cairo University Faculty of Medicine, Cairo, Egypt; 2 Department of Orthopaedic Surgery, Tanta University Faculty of Medicine, Tanta, Egypt; 3 Department of Orthopaedic Surgery, Menoufia University Faculty of Medicine, Shebin El-Kom, Menoufia, Egypt

**Keywords:** Proximal humeral tumors, Limb salvage, Reconstruction, Modular endoprosthesis, Cement spacer, Outcomes

## Abstract

**Background:**

There is no agreement about the best reconstructive option following resection of proximal humerus tumors. The purpose of this study was to compare the functional outcomes of endoprosthesis reconstruction versus nail cement spacer reconstruction after wide resection of proximal humeral tumors.

**Methods:**

This retrospective comparative study included 58 patients with proximal humerus tumors who had undergone tumor resection and reconstruction with modular endoprosthesis (humeral hemiarthroplasties) or cement spacer. Medical records were reviewed for the epidemiological, clinical, radiological, and operative data. Lung metastasis, local recurrence, and complication were also reviewed. The functional outcome was evaluated using the Musculoskeletal Tumor Society scoring (MSTS) system.

**Results:**

Nineteen patients with a mean age of 33.4 ± 17.5 years underwent reconstruction by modular endoprosthesis, and 39 patients with a mean age of 24.6 ± 14.3 years underwent reconstruction by cement spacer. The mean MSTS score was 24.8 ± 1.1 in the endoprosthesis group and 23.9 ± 1.4 in the spacer group, *P* = 0.018. Complications were reported in 5 (26.3%) patients in the endoprosthesis group and 11 (28.2%) patients in the spacer group, *P* = 0.879. There were no statistically significant differences in the functional outcomes in both patient groups with or without axillary or deltoid resection.

**Conclusions:**

Both endoprostheses and cement spacers are durable reconstructions with almost equal functional outcomes with no added advantage of the expensive endoprosthesis.

## Background

The proximal humerus is a frequent location of primary malignant tumors and metastatic bone disease [1]. In the past, the main treatment options for these tumors were amputation and shoulder disarticulation [2]. Currently, limb salvage surgery with reconstruction is the treatment of choice as it offers functional and cosmetic advantages, in addition to social and emotional patient acceptance [3].

Limb salvage surgery for these tumors is challenging to orthopedic oncologists and usually requires wide resection and subsequent reconstruction [4]. There is no agreement about the best reconstruction method, and the literature is limited regarding the potential variations in functional results and survival of different constructs [1, 4].

After extensive bone resection, different reconstructive options are employed to regain limb function [3, 5–9]. Each reconstructive method has its advantages and disadvantages [10, 11]. Factors that should be considered when evaluating a reconstruction method include the ease of the procedure, functional outcome, morbidity, complications, and durability [2, 4].

Most frequently utilized are tumor endoprosthesis, arthrodesis implants, autografts, allografts, and custom-made implants [3, 9]. Arthrodesis reconstruction makes patients rely on the scapulothoracic motion for performing daily living activities. Functional results are comparable between arthrodesis and motion-preserving reconstructions and between primary or secondary arthrodesis [12, 13].

Currently, the proximal humerus endoprosthesis is most commonly used after resecting the humerus with good functional outcomes, but it has the disadvantage of being expensive [1, 5–7, 9, 12, 14–18]. Nail cement spacer reconstruction is the alternative way to reconstruction that benefits from cost-effectiveness [3].

Tumor resections have significant challenges due to the need to sacrifice part of the deltoid and rotator cuff muscles in addition to the axillary nerve in most cases to achieve a wide margin. This leads to a compromise in the shoulder function and motion [12, 19]. Even with preserving the axillary nerve, sacrificing parts of the rotator cuff will lead to deltoid muscle malfunction and insufficiency of the abductor mechanism with limitation of the shoulder motion [20, 21].

We hypothesized that in the absence of sufficient abductor mechanism following tumor resection, any mobile reconstructive option would just act as a hanger with a limited range of motion. Hence, an expensive hanger will provide the exact function of an inexpensive one.

This study aimed to compare the functional and oncological outcomes of endoprosthesis reconstruction versus nail cement spacer reconstruction following wide resection of proximal humeral tumors.

## Methods

This comparative study was a prospective analysis of retrospective data of patients with primary malignant, benign aggressive, or metastatic tumors of the proximal humerus who had proximal humeral resection and reconstruction by modular tumor endoprosthesis (humeral hemiarthroplasties) or cement spacer. Surgeries were performed at a single orthopedic oncology center between September 2004 and December 2018. The minimum follow-up period was one year. Patients who did not complete the one-year follow-up, died of disease, or lost to follow-up, were excluded. Endoprosthesis reconstruction was in the form of humeral hemiarthroplasties and cement spacers were made of antibiotic-loaded polymethylmethacrylate (PMMA) augmented by a rush pin or Kuntscher nail inside it, acting as a stem. The study was conducted after approval from Menoufia University Institutional Review Board. Written informed consent was obtained from adult patients and from parents of patients below 16 years of age.

Medical records were reviewed for the epidemiological and clinical data, including the presentation, history of previous interventions, condition of the overlying skin, shoulder joint range of motion, and neurovascular examination.

Revision of available imaging studies, including full-length standard anteroposterior and lateral radiographs of the involved humerus, bone scintigraphy, chest computed tomography (CT), and magnetic resonance imaging (MRI) of the whole humerus was performed for assessment of the intramedullary and soft tissue extent of the tumor and relation of the tumor to the neurovascular structures. Lung metastasis was assessed in with chest CT scans.

Type of biopsy, whether open or closed, as well as neoadjuvant chemotherapy, were recorded. Histopathological reports following biopsy, as well as following tumor resection, were reviewed. Operative data included the approach, type of resection according to the classification system proposed by Malawer et al. [22], margin type, length of resection, reconstruction method, and implant type.

The functional outcomes were evaluated using the Musculoskeletal Tumor Society scoring (MSTS) system [23]. The postoperative shoulder range of motion was assessed. Any complications were recorded.

**Fig. 1:**
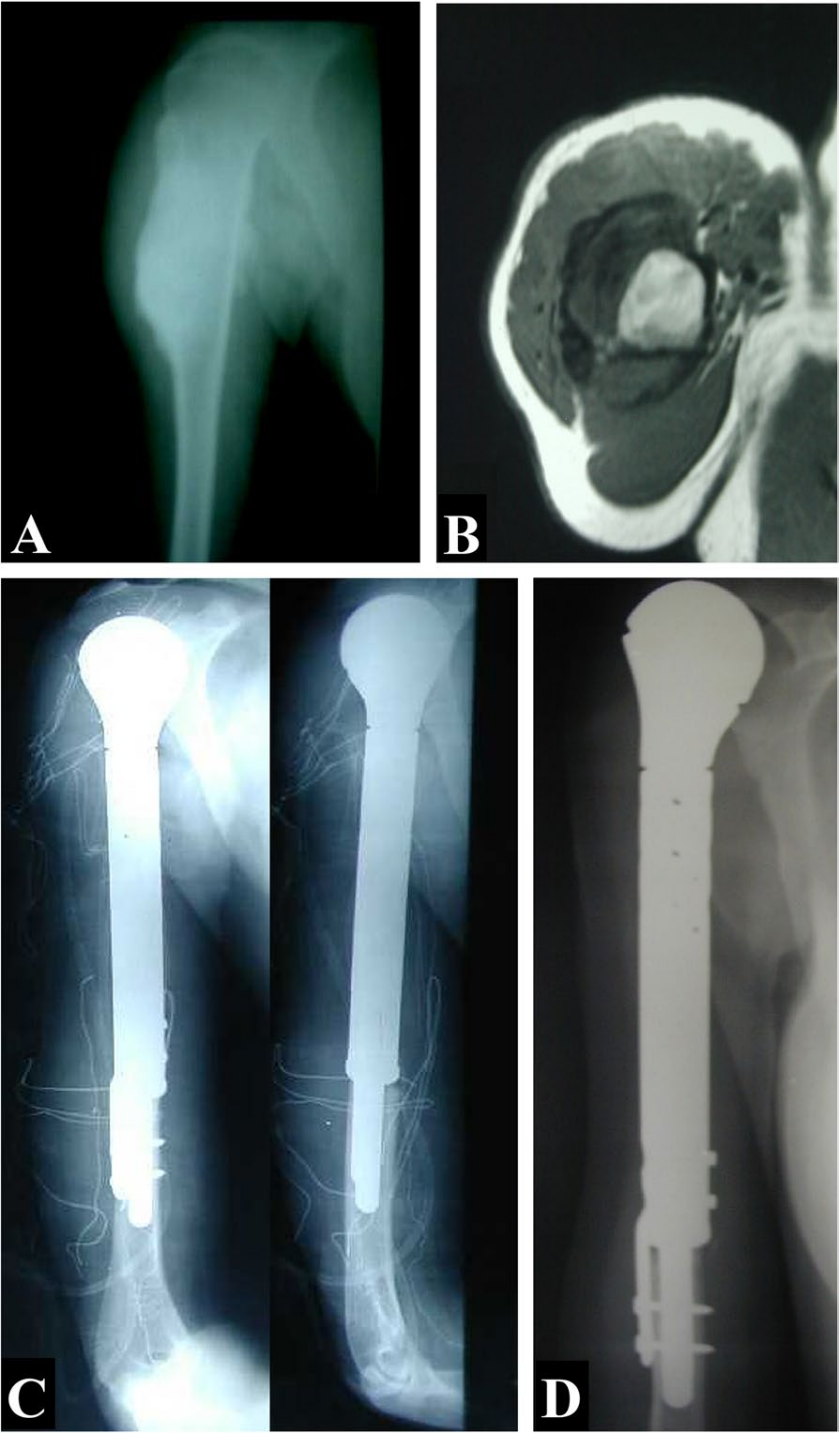
A 24-year-old female patient with osteosarcoma of the proximal humerus had undergone tumor resection and reconstruction with endoprosthesis. **A** Plain X-ray of the shoulder, anteroposterior view. **B** MRI axial view. **C** Postoperative X-rays, anteroposterior and lateral views. **D** Two-year follow-up X-rays, anteroposterior view

**Fig. 2:**
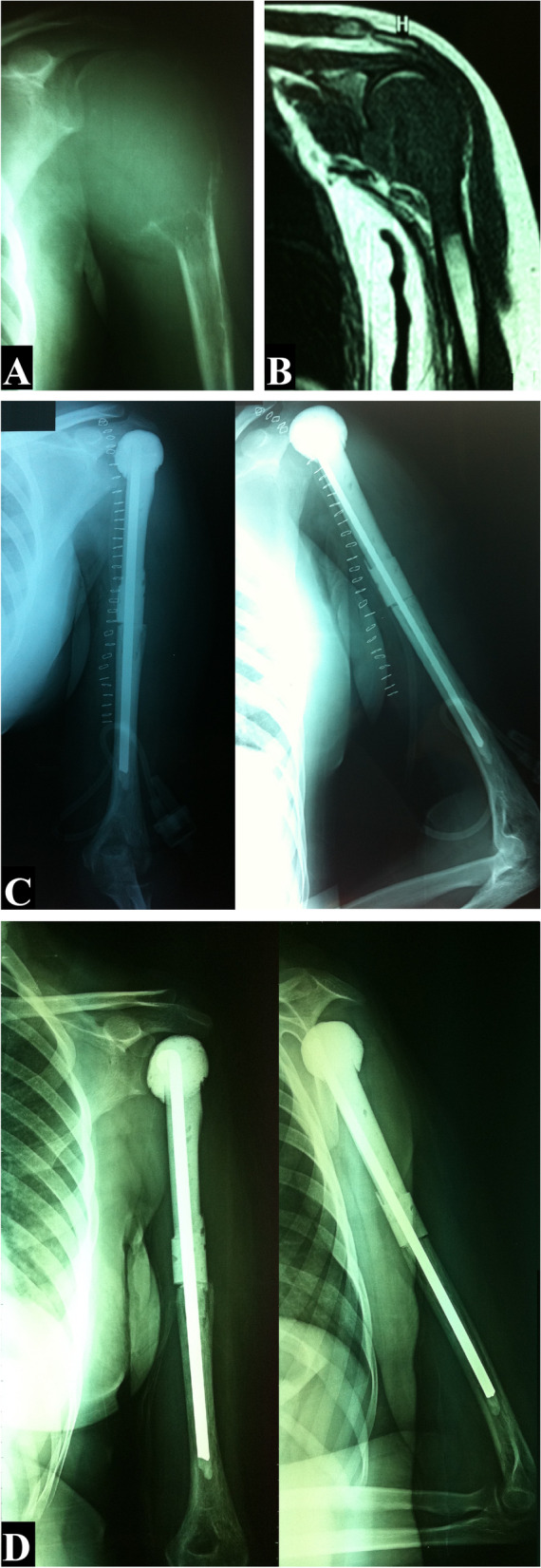
A 22-year-old female patient with giant cell tumor of the proximal humerus had undergone tumor resection and reconstruction with cement spacer. **A** Plain X-ray of the shoulder, anteroposterior view. **B** MRI sagittal view. **C** Postoperative X-rays, anteroposterior and lateral views. **D** Two-year follow-up X-rays, anteroposterior and lateral views

Oncological outcomes were also assessed, including local recurrence and lung metastasis. In addition, follow-up radiographs were evaluated for the implant position, any loosening, subluxation, or local recurrence, Figs. 1 and 2.

### Statistical analysis

IBM SPSS version 25.0 (SPSS Inc., Armonk, NY) was used for the data analysis. Categorical data were compared using the chi-square or Fisher’s exact tests, while continuous data were compared using the student’s t-test or Mann-Whitney U test. Statistical significance was set at a *P*-value of less than 0.05.

## Results

This study included 58 patients; 19 patients underwent reconstruction by modular endoprosthesis, while 39 patients underwent reconstruction by cement spacer. The endoprosthesis group included 9 (47.4%) males, and 10 (52.6%) females, with a mean age of 33.4 ± 17.5 (range, 13–77) years. The cement spacer group included 26 (66.7%) males, and 13 (33.3%) females, with a mean age of 24.6 ± 14.3 (range, 5–61) years, *P* = 0.061 and 0.159 for age and gender, respectively.

The most common diagnosis in the endoprosthesis group was chondrosarcoma (*n* = 7, 36.8%), followed by osteosarcoma (*n* = 5, 26.3%), while in the spacer group it was osteosarcoma (*n* = 17, 43.6%), followed by Ewing’s sarcoma (*n* = 12, 30.8%), *P* = 0.008.

Three (15.8%) and 8 (20.5%) patients were presented as recurrent cases in the endoprosthesis and spacer groups, respectively, *P* = 0.738. Seven (36.8%) and 11 (28.2%) patients were presented with a pathological fracture in the endoprosthesis and the spacer groups, respectively, *P* = 0.505, Table 1.

**Table 1:** Baseline and demographic data of the included patients

Data	Endoprosthesis group (***n*** = 19)		Cement spacer group (***n*** = 39)		***P***-value
	No.	%	No.	%	
**Age** (y)					
Mean ± SD	33.4 ± 17.5		24.6 ± 14.3		0.061
**Gender**					0.159
Male	9	47.4%	26	66.7%	
Female	10	52.6%	13	33.3%	
**Diagnosis**					**0.008**
Osteosarcoma (*n* = 22)	5	26.3%	17	43.6%	
Ewing’s sarcoma (*n* = 13)	1	5.3%	12	30.8%	
Chondrosarcoma (*n* = 12)	7	36.8%	5	12.8%	
Giant cell tumor (*n* = 5)	2	10.5%	3	7.7%	
Chondroblastoma (*n* = 2)	2	10.5%	0	0%	
Leiomyosarcoma (*n* = 1)	1	5.3%	0	0%	
Primary lymphoma (*n* = 1)	0	0%	1	2.6%	
Malignant fibrous histiocytoma (*n* = 1)	1	5.3%	0	0%	
Metastatic adenocarcinoma (*n* = 1)	0	0%	1	2.6%	
**Recurrent at presentation**					0.738
De novo (*n* = 47)	16	84.2%	31	79.5%	
Recurrent (*n* = 11)	3	15.8%	8	20.5%	
**Pathological fracture at presentation**					0.505
No (*n* = 40)	12	63.2%	28	71.8%	
Yes (*n* = 18)	7	36.8%	11	28.2%	

### Operative results

The most commonly used approach was the anteromedial approach, 13 (68.4%) and 29 (74.4%) patients, followed by the deltopectoral, 6 (31.6%), and 8 (20.5%), in the endoprosthesis and spacer groups, respectively, *P* = 0.784.

Regarding the type of resection, in the endoprosthesis group, it was type I (intra-articular proximal humeral resection) in 18 (94.7%) patients and type V (extra-articular humeral and glenoid resection) in one (5.3%) patient. In the spacer group, it was type I in 36 (92.3%) patients and type V in 3 (7.7%) patients, *P* = 0.999.

A wide margin was achieved in 18 (94.7%) patients in the endoprosthesis group and 38 (97.4%) patients in the spacer group, *P* = 0.999.

The mean length of resection was shorter in the endoprosthesis group, 13.7 ± 3.5 (range, 8–20) cm, than in the spacer group, 14.9 ± 3.7 (range, 9–26) cm, but the difference was statistically insignificant, *P* = 0.250.

A significant part of the deltoid (at least the anterior and the middle parts) was resected in 13 (68.4%) and 28 (71.8%) patients in the endoprosthesis and spacer groups, respectively, *P* = 0.791.

The axillary nerve was resected in 3 (15.8%) patients in the endoprosthesis and 20 (51.3%) patients in the spacer group, *P* = 0.010.

Additionally, the musculocutaneous nerve was resected in one patient in the endoprosthesis group and two patients in the spacer group. The radial nerve was resected in two patients in the spacer group.

The operative time was not different between the two groups, 3.3 ± 0.9 (range, 2–5) hours in the endoprosthesis group and 3.4 ± 0.9 (range, 2–6) hours in the spacer group, *P* = 0.958, Table 2.

**Table 2:** Operative details of the included patients

Data	Endoprosthesis group (***n*** = 19)		Cement spacer group (***n*** = 39)		***P***-value
	No.	%	No.	%	
**Approach**					0.784
Anteromedial (*n* = 42)	13	68.4%	29	74.4%	
Deltopectoral (*n* = 14)	6	31.6%	8	20.5%	
Anterior (*n* = 1)	0	0%	1	2.6%	
Posterior (*n* = 1)	0	0%	1	2.6%	
**Type of resection**					0.999
Type I (*n* = 54)	18	94.7%	36	92.3%	
Type V (*n* = 4)	1	5.3%	3	7.7%	
**Margin**					0.999
Wide margin (*n* = 56)	18	94.7%	38	97.4%	
Marginal margin (*n* = 2)	1	5.3%	1	2.6%	
**Length of resection (cm)**					
Mean ± SD	13.7 ± 3.5		14.9 ± 3.7		0.250
**Deltoid resection**					0.791
Resected (*n* = 41)	13	68.4%	28	71.8%	
Not resected (*n* = 17)	6	31.6%	11	28.2%	
**Axillary nerve resection**					**0.010**
Resected (*n* = 23)	3	15.8%	20	51.3%	
Not resected (*n* = 35)	16	84.2%	19	48.7%	
**Operative time** (hour)					
Mean ± SD	3.3 ± 0.9		3.4 ± 0.9		0.958

### Functional outcomes

The mean follow-up period was 79 ± 57 (range, 15–168) months in the endoprosthesis group, and 42.4 ± 36 (range, 12–149) months in the spacer group, *P* = 0.020.

Overall, the mean MSTS score for all patients was 24.2 ± 1.4 (range, 22–27). The mean MSTS score in the endoprosthesis group was 24.8 ± 1.1 (range, 23–27) points, while it was 23.9 ± 1.4 (range, 22–27) points in the spacer group, *P* = 0.018.

The mean MSTS score was 24.1 ± 1.3 points in patients who had deltoid resection and 24.5 ± 1.5 points in patients without deltoid resection, *P* = 0.384.

The mean MSTS score was lower in patients with axillary nerve resection than in patients with preserved axillary nerve, 23.7 ± 1.2 and 24.6 ± 1.6 points, respectively, *P* = 0.019.

When analyzing each group separately, there were no statistically significant differences in the functional outcomes in patients with or without axillary or deltoid resection, Table 3.

**Table 3:** Comparison of the functional outcome in patients with and without deltoid and axillary resection in both groups

Data	Endoprosthesis	***P***-value	Cement spacer	***P***-value
	MSTS score Mean ± SD		MSTS score Mean ± SD	
**Deltoid resection**		0.640		0.236
Resected	24.9 ± 1.2		23.8 ± 1.2	
Not resected	24.7 ± 0.8		24.4 ± 1.9	
**Axillary nerve resection**		0.766		0.097
Resected	24.7 ± 0.6		23.6 ± 1.2	
Not resected	24.9 ± 1.1		24.3 ± 1.6	

At the latest follow-up, the mean active shoulder forward flexion was 30.3 ± 35.8 (range, 0° – 140°) and 17.3 ± 20.0° (range, 0° – 70°) in the endoprosthesis and spacer groups, respectively, *P* = 0.184. Four patients were able to achieve forward flexion of more than 60° (two in each group). The mean active shoulder extension was 48.0 ± 25.3° (range, 30° – 90°) and 16.6 ± 18.7° (range, 0° – 60°) in the endoprosthesis and spacer groups, respectively, *P* = 0.075. The mean active shoulder abduction was 33.4 ± 18.7° (range, 10° – 90°) and 16.6 ± 18.7° (range, 0° – 70°) in the endoprosthesis and spacer groups, respectively, *P* = 0.006. Three patients achieved more than 60° of abduction (two in the endoprosthesis group and one in the spacer group), Table 4.

**Table 4:** Comparison of outcomes and complications in both groups

Data	Endoprosthesis group (***n*** = 19)		Cement spacer group (***n*** = 39)		***P***-value
	No.	%	No.	%	
**Follow-up period** (m)					
Mean ± SD	79 ± 57		42.4 ± 36		**0.020**
**MSTS score**					
Mean ± SD	24.8 ± 1.1		23.9 ± 1.4		**0.018**
**Active range of motion (°)**					
Forward flexion	30.3 ± 35.8		17.3 ± 20.0		0.184
Extension	48.0 ± 25.3		26.7 ± 19.1		0.075
Abduction	33.4 ± 18.7		16.6 ± 18.7		**0.006**
**Local recurrence** (*n* = 10)	2	10.5%	4	10.3%	0.975
**Chest metastasis** (*n* = 9)	3	15.8%	6	15.4%	0.968
**Complications**					0.879
Radial nerve palsy (*n* = 3)	0	0%	3	7.7%	
Deep infection (*n* = 2)	1	5.3%	1	2.6%	
Skin sloughing (*n* = 1)	0	0%	1	2.6%	
Wound gapping (*n* = 1)	0	0%	1	2.6%	
Implant failure and revision (*n* = 3)	1	5.3%	2	5.1%	
Dislocation (*n* = 1)	0	0%	1	2.6%	
Proximal migration (*n* = 4)	2	10.5%	2	5.1%	
Downward subluxation (*n* = 2)	1	5.3%	1	2.6%	

*MSTS* The Musculoskeletal Tumor Society

In patients with axillary nerve resection (*n* = 23) and those with spared axillary nerve (*n* = 35), there was no statistically significant difference in outcomes and range of motion between endoprosthesis and spacer in either group. In patients with deltoid resection, the functional outcome and range of extension and abduction were significantly higher in the endoprosthesis group, *P* = 0.007, 0.044, and 0.002, respectively, Table 5.

**Table 5:** Comparison of outcomes and range of motion between endoprosthesis and cement spacer in patients with and without axillary nerve and deltoid resection

Patients with axillary nerve resection (***n*** = 23)			
**Data**	**Endoprosthesis group (** ***n*** **= 3)**	**Cement spacer group (** ***n*** **= 20)**	***P*** **-value**
	Mean ± SD	Mean ± SD	
**MSTS score**	24.7 ± 0.6	23.6 ± 1.2	0.131
**Active range of motion (°)**			
Forward flexion	20.0 ± 14.1	8.0 ± 12.3	0.243
Extension	14.9 ± 17.9	15.0 ± 18.7	0.587
Abduction	20.0 ± 14.1	8.00 ± 12.3	0.243
**Patients without axillary nerve resection (** ***n*** **= 35)**			
**Data**	**Endoprosthesis group (** ***n*** **= 16)**	**Cement spacer group (** ***n*** **= 19)**	***P*** **-value**
	Mean ± SD	Mean ± SD	
**MSTS score**	24.9 ± 1.1	24.3 ± 1.6	0.252
**Active range of motion (°)**			
Forward flexion	31.9 ± 38.7	25.0 ± 22.4	0.590
Extension	48.0 ± 25.3	35.0 ± 15.5	0.248
Abduction	35.4 ± 18.9	35.0 ± 15.5	0.147
**Patients with deltoid resection (** ***n*** **= 41)`**			
**Data**	**Endoprosthesis group (** ***n*** **= 13)**	**Cement spacer group (** ***n*** **= 28)**	***P*** **-value**
	Mean ± SD	Mean ± SD	
**MSTS score**	24.9 ± 1.2	23.8 ± 1.2	**0.007**
**Active range of motion (°)**			
Forward flexion	24.1 ± 21.5	12.0 ± 18.2	0.134
Extension	55.0 ± 29.5	24.3 ± 18.8	**0.044**
Abduction	31.4 ± 12.7	11.0 ± 15.6	**0.002**
**Patients without deltoid resection (** ***n*** **= 17)**			
**Data**	**Endoprosthesis group (** ***n*** **= 6)**	**Cement spacer group (** ***n*** **= 11)**	***P*** **-value**
	Mean ± SD	Mean ± SD	
**MSTS score**	24.7 ± 0.8	24.4 ± 1.9	0.712
**Active range of motion (°)**			
Forward flexion	47.5 ± 62.4	28.6 ± 20.4	0.466
Extension	37.5 ± 15.0	30.0 ± 21.2	0.571
Abduction	38.0 ± 29.5	28.6 ± 20.4	0.525

*MSTS* The Musculoskeletal Tumor Society

Overall, local recurrence occurred in 6 patients; 2 patients in the endoprosthesis group, both were diagnosed as chondrosarcoma, and 4 patients in the spacer group; osteosarcoma (*n* = 3) and Ewing’s sarcoma (*n* = 1), *P* = 0.975.

Lung metastasis was recorded in 8 patients; 3 patients in the endoprosthesis group; all were diagnosed with chondrosarcoma, and 5 patients in the spacer group; osteosarcoma (*n* = 4), Ewing’s sarcoma (*n* = 1), *P* = 0.758, Table 4.

### Complications

Overall, complications occurred in 5 (26.3%) patients in the endoprosthesis group and 11 (28.2%) patients in the spacer group, *P* = 0.879.

Three patients developed radial nerve palsy following nerve resection with the tumor; all were in the spacer group. Two patients had a deep infection, one in the endoprosthesis group, treated by debridement and insertion of cement spacer, and one in the spacer group managed by debridement and spacer revision. Skin sloughing occurred in one patient in the spacer group and was treated by debridement. Wound gapping occurred in one patient in the spacer group and was treated by secondary sutures. One patient in the spacer group had anterior dislocation 10 months postoperatively and was managed by open reduction. Two patients in the endoprosthesis group and two in the spacer group had proximal migration of prosthesis treated conservatively. One patient in the endoprosthesis group and one in the spacer group had downward subluxation treated conservatively. Two patients in the spacer group had broken rush pin, which was revised by intramedullary nail, Table 4.

The 3-year survival rates were 94.7 and 94.9% for the endoprosthesis and spacer groups, respectively, *P* = 0.592.

## Discussion

Limb salvage surgery rather than amputation has become the ideal management option for malignant tumors of the proximal humerus, providing functional and cosmetic benefits [24, 25]. Several reconstructive options are available following tumor resection, including autologous grafts, osteoarticular allografts, endoprosthesis, allograft–prosthesis composite, or nail cement spacer [7, 9, 16, 26].

Resection of proximal humeral tumors often includes resection of parts of the deltoid and rotator cuff muscles as well as the axillary nerve, which impacts the expected shoulder function following reconstruction [20]. In our study, soft tissue reconstruction and attachment of remaining muscles to each other (for example, deltoid to pectoralis major, capsule and rotators to the implant, deltoid remnants to short biceps, and short biceps to long biceps) and to the prosthesis or spacer was aided by Ethibond and FiberWire sutures that were wrapped around the prosthesis or passed through the cement spacer before the setting of cement. Other methods of attachments were proline mesh and trevira tube to provide better anchorage of muscles, sometimes providing a sling to the acromion. Coverage of the implant or the spacer was the primary aim of avoiding dead space, even in case of inclusion of axillary nerve in the resection.

In this study, we compared the functional and oncological outcomes, complications, and survival rates of the endoprosthesis reconstruction versus nail cement spacer reconstruction following wide resection of proximal humeral tumors.

The mean follow-up period of the endoprosthesis group was significantly longer than the spacer group as we originally used to do only endoprosthesis. Over time we became more selective, reserving endoprosthesis for patients with minimal muscle resections, preserved axillary nerve, and potentially less expected functional deficit. Accordingly, most of the patients in the endoprosthesis group had chondrosarcoma, whereas the commonest diagnosis in the spacer group was osteosarcoma, followed by Ewing’s sarcoma. Moreover, the mean age in the spacer group was lower, as the group included many children in which the spacer was more appropriate and available to match the size of the humeral medulla as well as the glenoid dimensions. Both reconstructive techniques had almost equal operative time.

The average MSTS score for all patients in this study was 24.2 (80%). This was similar to other reports. Van de Sande et al. [16] compared the functional outcomes of proximal humeral reconstructions with modular tumor prosthesis, osteoarticular allograft, and allograft-prosthesis composite in 37 patients after tumor resection with a mean follow-up of 10 years. The average MSTS scores were 77% for the endoprosthesis reconstruction, 76% for the osteoarticular allograft, and 72% for the allograft-prosthetic composite groups; however, endoprosthesis reconstruction had the highest implant survival and the lowest complication rate [16]. A systematic review by Teunis et al. [27] compared the functional outcomes of different reconstruction methods, and the average MSTS scores ranged from 61 to 77% in the prostheses, 50 to 78% in the osteoarticular grafts, and from 57 to 91% the allograft-prosthesis composites studies. The conclusion was that both endoprosthesis reconstruction and allograft-prosthesis composites have comparable functional outcomes and survival rates, with avoidance of fractures observed with osteoarticular allografts [27].

Studies reporting on the outcome of spacers had inferior results. Kundu et al. [1] treated 14 patients with nail cement spacer reconstruction after tumor excision of the proximal humerus and reported a mean MSTS score of 19.09 with a mean follow-up of 30.14 months. Singh et al. [28] reported two cases with Ewing’s sarcoma and metastatic tumor of the proximal humerus treated with radical excision and cement spacer reconstruction and reported satisfactory shoulder, elbow, and hand function.

In our study, the endoprosthesis group had a slightly better functional outcome compared to the nail spacer group. Moreover, the shoulder range of motion was better in the endoprosthesis group. The active abduction, forward flexion, and extension were almost double, yet only the difference in the abduction was statistically significant. Rafalla and Abdullah [3] reported similar functional outcomes between endoprosthesis and spacers. In our study, the better functional outcome in the endoprosthesis group was probably due to selection bias as the endoprosthesis was done more in smaller tumors with lesser muscle resection and more patients with preserved axillary nerve.

When we divided patients into 2 groups; patients with axillary nerve resection and those with spared axillary nerve, there was no statistically significant difference in outcome between endoprosthesis and spacer in either group. Thus, the difference in function is probably influenced by the available muscles and nerves rather than the reconstructive modality.

If we excluded the patients with radial nerve palsy due to the inclusion of the radial nerve with the resected specimen, most of the complications were due to instability, dislocation, superior migration, or downward subluxation. There was no statistically significant difference in the complication rate between both groups. In our study, there was no dislocation in the endoprosthesis group and the dislocation rate in the spacer group was 2.6%. Our rates were lower than the rate of 7.5% reported in Scotti et al. [29], who treated 40 patients with proximal humeral metastasis with endoprosthesis reconstruction. Rafalla and Abdullah [3] reported subluxation in one (12.5%) case out of 8 cases that had endoprosthesis reconstruction. Potter et al. [9] reported a 31% (5 out of 16 patients) rate of subluxation or dislocation following endoprosthesis reconstructions of the proximal humerus.

Only 2 patients in this study developed infection, one in each group. The endoprosthesis was treated by debridement, removal, and insertion of cement spacer, and the infected spacer was managed by debridement and spacer revision. Our study showed that both reconstructive techniques had similar durability and comparable 3-year survival rates.

Although the aim of this study was not to evaluate the oncological outcome of the patients with proximal humeral tumors, there was no statistically significant difference in local recurrence and lung metastases between the endoprosthesis and spacer groups.

Even with preserving the axillary nerve, sacrificing parts of the rotator cuff will lead to deltoid muscle malfunction and insufficiency of the abductor mechanism with limitation of the shoulder motion. Thus, in the absence of sufficient abductor mechanism following tumor resection, any mobile reconstructive option would just act as a hanger with a limited shoulder range of motion.

This study has some limitations, including its retrospective nature, selection bias, and the relatively low number of patients in each group. However, the study was done in one institution by the same surgeons and with a reasonable follow-up. Longer periods of follow-up will test the durability of both reconstructive options.

## Conclusions

Both endoprostheses and spacers are durable reconstructions that will provide the patient with almost equal functional outcomes. This outcome is better if the axillary nerve is preserved.

## Data Availability

The dataset analyzed in this study is available from the corresponding author on reasonable request.
